# Supplementary figures and images for: Multiple cyanotoxin congeners produced by sub-dominant cyanobacterial taxa in riverine cyanobacterial and algal mats
Source: PLoS One. 2019 Dec 16;14(12):e0220422. doi: 10.1371/journal.pone.0220422 (PMC6913960; doi:10.1371/journal.pone.0220422)

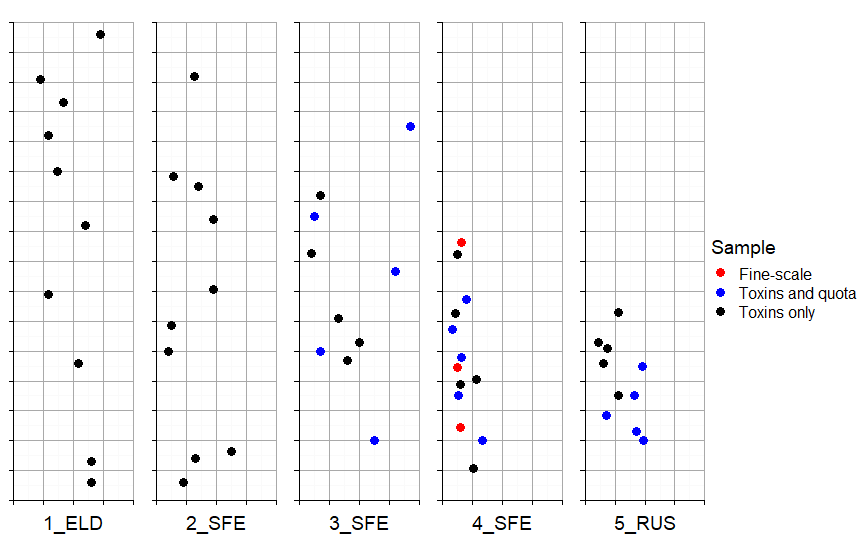

Supplement: S1 Fig — Squares in the grids represent 1 m2. Fine-scale samples consisted of five samples collected from periphyton on a single cobble. (TIFF) [file pone.0220422.s001.tiff]
