# Supplementary material for: Multiple cyanotoxin congeners produced by sub-dominant cyanobacterial taxa in riverine cyanobacterial and algal mats
Source: PLoS One. 2019 Dec 16;14(12):e0220422. doi: 10.1371/journal.pone.0220422 (PMC6913960; doi:10.1371/journal.pone.0220422)
Supplement: S1 Table — (DOCX) [file pone.0220422.s002.docx]

**S1 Table.** Primers and probes used for PCR screening for cyanotoxin genes and for ddPCR analysis of *anaC* gene copy number.

| **Toxin** | **Target Gene** | **Reaction** | **Primer and probe sequences** | **Reference** |
| --- | --- | --- | --- | --- |
| Anatoxins | *anaC* | PCR  Sequencing | *anaC-gen-F*  5′-TCTGGTATTCAGTCCCCTCTAT-3′  *anaC-gen-R*  5′-CCCAATAGCCTGTCATCAA-3′ | Rantala-Ylinen, Känä (25) |
| Anatoxins (*Oscillatoria*/*Microcoleus autumnalis*-specific) | *anaC* | ddPCR | *Phor-AnaC-F5*  5′-ACTAACCGAATCACTTCCACTT-3′  *Phor-AnaC-R5*  5′-CTCACCCACCTCACCTTTAG-3′  *Phor-AnaC-P5*  5′-TTCAGTATTAGCGCAGGCTTTGCC-3′ | Kelly, Wood (20) |
| Microcystin  Nodularin | *mcyE*  *ndaF* | PCR  Sequencing | *HEPF*  5′-TTTGGGGTTAACTTTTTTGGGCATAGTC-3′  *HEPR*  5′-AATTCTTGAGGCTGTAAATCGGGTTT-3′ | Jungblut and Neilan (23) |
| Cylindrospermopsin | *cyrJ* | PCR | *cynsulfF*  5′-ACTTCTCTCCTTTCCCTATC-3′  *cylnamR*  5′-GAGTGAAAATGCGTAGAACTTG-3′ | Mihali, Kellmann (26) |
| Saxitoxin | *sxtA* | PCR | *Sxtaf*  5′-GCGTACATCCAAGCTGGACTCG-3′  *Sxtar*  5′-GTAGTCCAGCTAAGGCACTTGC-3′ | Ballot, Fastner (24) |
