# Supplementary material for: Multiple cyanotoxin congeners produced by sub-dominant cyanobacterial taxa in riverine cyanobacterial and algal mats
Source: PLoS One. 2019 Dec 16;14(12):e0220422. doi: 10.1371/journal.pone.0220422 (PMC6913960; doi:10.1371/journal.pone.0220422)
Supplement: S2 Table — (DOCX) [file pone.0220422.s003.docx]

**S3 Table.** The mean proportion of each anatoxin congener from attached periphyton samples collected at the five sites ± standard deviation (n = 10 for all sites except 5_RUS where n = 9).

| **Site** | **ATX** | **dhATX** | **HTX** | **dhHTX** |
| --- | --- | --- | --- | --- |
| 1_ELD | 50.7 ± 15.8 | 38.7 ± 15.4 | 6.5 ± 7.8 | 4.1 ± 6.3 |
| 2_SFE | 56.5 ± 15.7 | 39.7 ± 15.5 | 0 ± 0 | 3.8 ± 6.1 |
| 3_SFE | 60.5 ± 15.5 | 39.4 ± 15.5 | 0.02 ± 0.51 | 0.02 ± 0.48 |
| 4_SFE | 28.5 ± 14.3 | 71.5 ± 14.3 | 0 ± 0 | 0.06 ± 0.79 |
| 5_SFE | 51.4 ± 15.8 | 48.6 ± 15.8 | 0 ± 0 | 0.06 ± 0.79 |
